# Assessing the Role of Pericardial Fat as a Biomarker Connected to Coronary Calcification—A Deep Learning Based Approach Using Fully Automated Body Composition Analysis

**DOI:** 10.3390/jcm10020356

**Published:** 2021-01-19

**Authors:** Lennard Kroll, Kai Nassenstein, Markus Jochims, Sven Koitka, Felix Nensa

**Affiliations:** 1 Institute of Diagnostic and Interventional Radiology and Neuroradiology, University Hospital Essen, 45147 Essen, Germany; kai.nassenstein@uk-essen.de (K.N.); sven.koitka@uk-essen.de (S.K.); felix.nensa@uk-essen.de (F.N.); 2 Institute of Artificial Intelligence in Medicine, University Hospital Essen, 45147 Essen, Germany; 3 Department of Radiology, Elisabeth-Krankenhaus Essen, 45138 Essen, Germany; 4 Cardiologic Practice Ratingen, 40882 Ratingen, Germany; m.jochims@kardiologie-ratingen.de

**Keywords:** epicardial adipose tissue, paracardial adipose tissue, body composition analysis, deep learning, artificial intelligence, atherosclerosis

## Abstract

(1) Background: Epi- and Paracardial Adipose Tissue (EAT, PAT) have been spotlighted as important biomarkers in cardiological assessment in recent years. Since biomarker quantification is an increasingly important method for clinical use, we wanted to examine fully automated EAT and PAT quantification for possible use in cardiovascular risk stratification. (2) Methods: 966 patients with intermediate Framingham risk scores for Coronary Artery Disease referred for coronary calcium scans were included in clinical routine retrospectively. The Coronary Artery Calcium Score (CACS) was extracted and tissue quantification was performed by a deep learning network. (3) Results: The Computed Tomography (CT) segmentations predicted by the network indicated no significant correlation between EAT volume and EAT radiodensity when compared to Agatston score (r = 0.18, r = −0.09). CACS 0 category patients showed significantly lower levels of total EAT and PAT volumes and higher EAT and PAT densities than CACS 1–99 category patients (*p* < 0.01). Notably, this difference did not reach significance regarding EAT attenuation in male patients. Women older than 50 years, thus more likely to be postmenopausal, were shown to be at higher risk of coronary calcification (*p* < 0.01, OR = 4.59). CACS 1–99 vs. CACS ≥100 category patients remained below significance level (EAT volume: *p* = 0.087, EAT attenuation: *p* = 0.98). (4) Conclusions: Our study proves the feasibility of a fully automated adipose tissue analysis in clinical cardiac CT and confirms in a large clinical cohort that volume and attenuation of EAT and PAT are not correlated with CACS. Broadly available deep learning based rapid and reliable tissue quantification should thus be discussed as a method to assess this biomarker as a supplementary risk predictor in cardiac CT.

## 1. Introduction

Epicardial Adipose Tissue (EAT) and Paracardial Adipose Tissue (PAT) are considered to be metabolically active organs surrounding the heart and adjacent vessels. In recent years, various studies indicated that EAT is linked to cardiovascular pathologies such as coronary artery disease, atrial fibrillation, and heart failure with preserved ejection fraction and future major adverse cardiac events (MACE) [1,2,3]. While obesity might be a driving factor in enhancing deranged adipogenesis in EAT and result in secretion of proinflammatory cytokines [4,5], other hormonal factors like diabetes mellitus or menopause seem to influence these two fat depots in their paracrine activity on the cardiovascular system [6,7]. These pathological developments in pericardial fat depots can be measured and quantified by Computed Tomography (CT).

### 1.1. Distinguishing Epicardial and Paracardial Adipose Tissue

Defining epicardial, paracardial and pericardial adipose tissue is of great relevance since these tissues differ in their embryological descent and function. While the term Pericardial Adipose Tissue encompasses both Epi- and Paracardial Adipose Tissue [8], the distinction between the latter two tissues has to be stressed. As Iacobellis et al. [9] described, EAT is defined as visceral fat located below the parietal pericardium surrounding the myocardium with no dividing fascia to the latter, which explains the shared microcirculation [10]. As EAT increases, it progressively fills the space between the ventricles and the remaining epicardial surface. EAT can also extend into the myocardium, following the adventitia of the coronary artery branches [9]. PAT is defined as the fat deposit in the mediastinum adjacent to the parietal pericardium: it surrounds the perivascular space of the adventitia of the coronary arteries, as well as the space outside the visceral pericardium and on the external surface of the parietal pericardium [11].

From an embryological perspective, the distinction is clear: EAT evolves from brown adipose tissue during embryogenesis, likewise from omental and mesenteric fat cells, and derives from the splanchnopleuric mesoderm [9,12]. In contrast to PAT, EAT is innervated and contains stromal, inflammatory, and immune cells [4]. PAT derives from the primitive thoracic mesenchyme, which splits into two layers, forming the parietal pericardium from the inner layer and the outer thoracic wall from the outer layer [9]. Therefore, PAT is perfused by branches of the internal thoracic artery, like the pericardiacophrenic artery [9].

### 1.2. EAT and the Relationship to Coronary Calcification

The anatomical proximity of EAT to the myocardium and coronary arteries results in various paracrine effects with which EAT seems to modulate heart function and inflammatory processes [13]. However, it is also considered a risk factor for atherosclerosis and atrial fibrillation [1,14]. Goeller et al. described significantly higher EAT volume in patients with coronary calcium, especially at an early stage of atherosclerosis, compared to patients without coronary calcification [3]. Furthermore, EAT radiodensity was significantly lower in patients with coronary calcium, which might be related to a higher cardiovascular risk [3,15]. Eisenberg et al. examined EAT and Coronary Artery Calcium Score (CACS) on the EISNER trial, showing that EAT and CACS were independent future predictors of MACE, and that EAT was a predictor of such events in patients with no coronary artery calcification (CAC) [16].

### 1.3. PAT Accumulation as a Potential Sign of Cardiovascular Risk in Postmenopausal Women

PAT is currently not considered to have a similar impact on Coronary Artery Disease (CAD) as EAT [17]. Still, PAT might play a more specific role in different respects: El Khoudary et al. suggests that PAT could be a “potential menopause-specific coronary artery disease risk marker” [7]. In multiple studies, an association between PAT accumulation and increased coronary calcium presence could be shown, with the menopausal stage and estradiol and hormone therapy accounted for [7,18].

### 1.4. AI for Clinical Biomarker Extraction

Artificial Intelligence is an emerging tool for biomarker extraction and precisely quantifying biomarkers in large-scale study cohorts [19,20,21]. Some deep learning-based (DL) approaches to EAT have already been demonstrated [16,21]. While CT scans are performed for various clinical indications, potentially valuable biometric data often goes unused. Body composition analysis (BCA) enables the quantification of biomarkers and processing of biometric data.

To our knowledge, there are currently no studies that have integrated epicardial and paracardial fatty tissue quantification into a fully automated body composition analysis.

In the present study, using fully-automated body composition analysis, we investigated the statistical correlation between epi- and paracardial adipose tissue and calcification score in cardiac CT in a relatively large cohort of patients from clinical routine care.

## 2. Experimental Section

### 2.1. Study Design and Study Population

In this retrospective study, 966 consecutive outpatients with either increased cardiovascular risk factors or atypical chest pain referred for a coronary calcium CT scan at the Radiology Department of the Elisabeth Krankenhaus Essen between June 2013 and January 2020 were included. Pursuant to German clinical practice recommendations, patients with Framingham risk scores indicative of an intermediate CAD risk were referred for coronary calcium CT scans [22,23,24]. 632 of the 996 patients were female, and 334 were male. Standardized cardiac CT scans were obtained for every patient. Non-contrast-enhanced CT scans amounted to 741 scans (35% male/65% female), and contrast-enhanced CT scans accounted for 225 scans (34% male/66% female). Table 1 and Table 2 outline the characteristics of the study population and the performed examinations.

### 2.2. Dual Source Computed Tomography Examinations and Coronary Calcium Scoring

Dual Source Computed Tomography (DSCT) examinations were conducted using a 2 × 128 detector row DSCT scanner (SOMATOM Definition Flash, Siemens Medical Solutions, Forchheim, Germany) with a gantry rotation time of 280 ms and a temporal resolution of 75 ms (collimation: 128 × 0.6 mm, slice thickness: 5 mm, reconstruction increment: 2.5 mm, tube current time product per rotation: 80 mAs, tube voltage: 120 kV). CARE Dose4D™ algorithm (Siemens Medical Solutions, Forchheim, Germany) was applied to minimize radiation exposure. The tube current modulation algorithm “ECG-pulsing” (Siemens Medical Solutions, Forchheim, Germany) was used, resulting in a dose reduction of 80% outside the pulsing window, which was set between 50% and 70% of the R–R interval. The radiation dose was estimated by the dose-length product and the Computed Tomography Dose Index volume (CDTIvol) according to the European Working Group for Guidelines on Quality Criteria in CT [25]. All scans were performed in craniocaudal direction during inspiratory breath-hold with reconstruction at 60% of the R–R interval. All scans were evaluated using dedicated software Syngo.Via VB 30 (Siemens Medical Solutions, Erlangen, Germany). Coronary calcifications were defined on CT images as the presence of more than two adjacent pixels with Hounsfield units greater than 130. The Agatston score was calculated automatically depending on the extent and density of the coronary lesions [26]. Additionally, the volumetric score was calculated by automatic multiplication of the calcified area in axial slices by slice thickness. Coronary Artery Calcium Score (CACS) was reported in three categories: no coronary calcium (CACS of 0), mild CAC (CACS 1–99) and more advanced CAC (CACS ≥100) [27,28] pursuant to common CACS grouping [24].

### 2.3. Deep Learning Architecture

For this study, an in-house DL-based body composition analysis system was utilized, which is an evolution from the system described in Koitka et al. [19]. Abdominal, thoracic, and head-and-neck CT scans, both non-contrast enhanced and contrast enhanced, can be processed in order to get tissue composition profiles for these CT scans.

The DL system utilizes a multi-resolution U-Net 3D network in order to segment the body into semantic regions. Adipose and muscular tissues are identified using known Hounsfield unit thresholds, −190 to −30 HU for adipose tissue and −29 to 150 HU for muscular tissue [29], and afterwards subclassified using the semantic body regions from the deep learning system. This approach allows to quantify five different adipose tissue biomarkers (subcutaneous adipose tissue, visceral adipose tissue, intermuscular adipose tissue, EAT and PAT) as well as the muscular tissue volume. Furthermore, segmented regions like the pericardium can also be used directly to measure the total volume enclosed by the pericardial sac. Technical details of the respective methodology is disclosed in Koitka et al. [19]. For this study, a training dataset of 100 thoracic CT scans divided 50/50 with (non-) contrast enhanced scans was pre-processed by three experienced human readers.

### 2.4. Quantification of Epicardial Adipose Tissue

The EAT examination was performed by a fully automated deep learning-based algorithm. Considering the fine differentiations necessary between these fat deposits, as stated in Section 1.1, the relevant regions were defined accordingly as follows: to extract measurements for EAT, the pericardium was segmented in the training dataset. EAT was derived from the pericardium region via automated HU thresholding for adipose tissue which was defined as any adipose voxels between −190 and −30 HU [19,29]. Based on the definition of Bertaso et al. [8], the mediastinum, confined by the superior and inferior thoracic aperture, was segmented and PAT was defined as any adipose voxel in this region.

Both fat depots were measured automatically into EATvol (EAT Volume) and EATatt (EAT Attenuation), as well as PATvol (PAT Volume) and PATatt (PAT Attenuation). Since cardiac CT imaging was confined within the superior and inferior borders of the heart before acquisition, due to dose reduction, PAT could not be measured in total.

### 2.5. Statistical Analysis

Statistical analysis was conducted using SciPy software [30] (version 1.5.4 for Linux). Continuous variables are stated as mean ± standard deviation (SD) and 95%-confidence interval (95%-CI), while categorical variables are stated as frequencies and percentages. Pearson rank correlations were calculated to determine the relationships between tissue type measures and the Agatston score, BMI, age, and gender. Because the pre-analysis of the dataset indicated not normally distributed data, the Kruskal-Wallis Test and post hoc Dunn’s test were performed to compare CACS categories and gender groups. Dunn’s test evaluates stochastic dominance and reports the results among multiple pairwise comparisons after a Kruskal-Wallis test evaluates stochastic dominance among k groups [31]. Kruskal-Wallis Test (KW-Test) results therefore indicate whether an incremental stochastic difference in a group is present, while the Dunn’s Test (DT) evaluates significant differences between subgroups of that category. The Odds Ratio was calculated to compare the strength of the statistical association between tissue characteristics and CACS in different sub-cohorts. A two-sided independent t-test was performed to compare two different age groups in the female sub-cohort. A *p*-value of <0.01 was considered statistically significant. The level of significance was set this high in order to take the large volume of included patients into account.

## 3. Results

### 3.1. Study Population

966 patients were included, summarized in detail in Table 1 and Table 2. 19 patients with insufficient data or motion artefacts were reviewed and excluded beforehand. Common CACS grouping was used as mentioned above.

### 3.2. DL-Network Performance in Tissue Quantification

**Figure 1:**
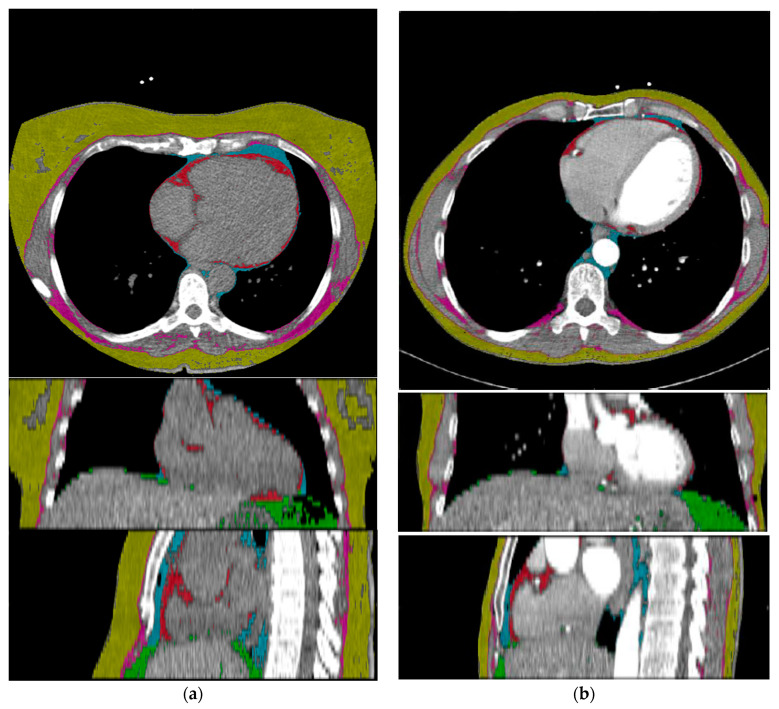
Exemplary adipose tissue predictions of a non-contrast-enhanced Chest CT (**a**) and a contrast-enhanced Chest CT scan (**b**). Description of labels and colors: yellow: subcutaneous adipose tissue, pink: intermuscular adipose tissue, cyan: PAT, red: EAT, green: visceral adipose tissue.

After training the deep learning system, it reached highly accurate predictions on CT scans of all tissue types on the independent test-dataset. The Sørensen Dice Score for relevant semantic body regions on the test set showed: thoracic cavity: 0.98, mediastinum: 0.90, pericardium: 0.96, subcutaneous tissue: 0.97 and muscle: 0.96. Subsequently, a fully automated BCA was performed on the complete study cohort using the trained system (Figure 1).

### 3.3. Measuring EAT and PAT in Relation to CACS

EATvol and PATvol, as well as EATatt and PATatt, were not significantly correlated to the individual Agatston scores (EATvol r = 0.18, EATatt r = −0.09, PATvol r = 0.25, PATatt r = −0.14).

**Figure 2:**
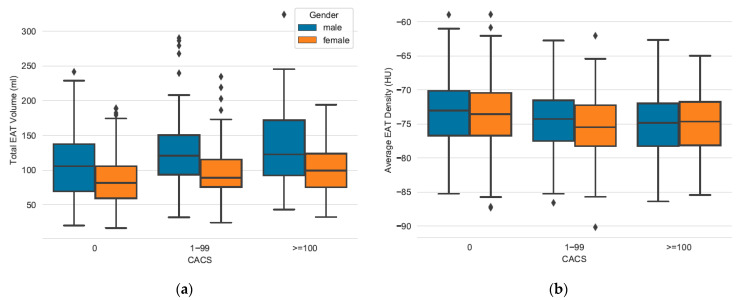
Comparing EATvol and EATatt between CACS categories and gender groups. (**a**) EAT volume increases significantly between CACS 0 vs. CACS 1–99 in women and men. No significance between CACS 1–99 vs. CACS ≥100 for both sexes. (**b**) EAT attenuation increases significantly between CACS 0 vs. CACS 1–99 in women, but not in men. No significance between CACS 1–99 vs. CACS ≥100 for both sexes (see Table 3).

After dividing data into CACS categories, the following observations can be made: CACS 0 category patients showed significantly lower levels of total EATvol than those with coronary calcium (CACS 0 vs. 1–99). However, patients with mild CAC (CACS 1–99) compared to patients with advanced CAC (CACS ≥100) did not show significant differences in EATvol levels, as shown in Figure 2. Table 3 outlines these differences in a group-wise comparison. When subdivided into gender-specific groups, women in CACS 1–99 category showed significantly higher levels of EATvol than those with no CAC, as well as men in these categories.

Similar observations could be made concerning EATatt. Patients with coronary calcification had lower levels of radiodensity in EAT compared to patients with no coronary calcification (Table 2). These categories showed significant differences when compared between CACS 0 vs. CACS 1–99. However, these categories did not show significant differences when compared between CACS 1–99 vs. CACS 100 categories (Figure 2). Itemized into gender groups, the described relation was significant for female patients, while it was not above significance level for male patients.

PAT was of significantly lower volume and of higher radiodensity in patients without CAC (CACS 0) compared to those with mild CAC (CACS 1–99). Mild CAC compared to advanced CAC (CACS ≥100) showed no significant differences in neither PATvol nor PATatt levels (Table 2 and Table 3). The described relation between CACS 0 and CACS 1–99 was significant for female patients, as well as for male patients with respect to PATvol and PATatt levels.

All these findings were independent from BMI, which did not increase or decrease significantly when comparing CACS categories.

Table 2 lists all the features measured, while Table 3 shows a complete inter-group comparison of CACS-groups.

### 3.4. Secondary Findings: Age Based Comparison of Female Patients between Pericardial Fat and CACS Categories

Age was a significant risk factor for CAC in both mild and advanced stages (see Table 3).

When examined using the independent two-sided *t*-test, women aged older than 50 years were significantly more likely to show more coronary calcification (CACS ≥10) than women younger than 51 years (*p* < 0.001, OR = 4.598). In the female sub-cohort consisting of 632 patients, 62.03% had no calcifications (compared to 40.11% of men), 26.42% were ranked CACS 1–99 (compared to 37.72% of men) and 11.55% were ranked CACS ≥100 (compared to 22.15% of men). However, no specific correlation could be observed when correlating these following parameters: the Pearson coefficient for PATvol and PATatt and to age was r = 0.391, r = −0.393 in female CACS 0 patients, r = 0.168 and r = −0.274 in female CACS 1–99 patients, and r = 0.178 and r = −0.229 in CACS ≥100 patients.

## 4. Discussion

In the present study, we used fully-automated body composition analysis to investigate the statistical correlation between epi- and paracardial adipose tissue and calcification score in cardiac CT in a relatively large cohort of patients from clinical routine care.

The volume and radiodensity of EAT and PAT do not correlate significantly with Agatston Score (EATvol r = 0.19, EATatt r = −0.09, PATvol r = 0.25, PATatt r = −0.14). Patients with mild CAC (CACS 1–99) showed significantly increased volumes of EAT and PAT in comparison to patients with no CAC (*p* < 0.01) but showed significantly decreased levels of EAT and PAT radiodensity in the same compared categories (*p* < 0.01). The DL-network performed well on the cohort dataset and successfully provided automated EAT and PAT assessment. The cohort is characterized by its intermediate Framingham risk score for CAD [24], resulting in the relative preponderance of female patients who are generally less prone to develop CAD compared to males. According to national clinical practice recommendations, these patients were referred for CT calcium scans in order to examine atypical symptoms. CT calcium scans have a high negative predictive value for these individuals to either rule out or prove the existence of CAC in order to intervene early in the pathogenesis of CAD [23].

Because EAT and PAT do not show a significant correlation when compared with CACS, these parameters can therefore not be used as a surrogate marker for CAC. No cut-off scores in EAT and PAT characteristics could be identified for high risk CAD. Although more female patients than male patients were included in this cohort and relatively more women compared to men had no CAC at all, significant differences in EAT and PAT characteristics between CACS 1–99 and CACS 0 were found in both subgroups. The observed sex-based difference in EAT accumulation requires further investigation. Moreover, women had higher odds of developing more coronary calcification (CACS ≥10) after reaching the mean age of menopause [32] compared to younger women, as laid out below. Remarkably, these findings were independent of BMI, which might indicate that these two fat deposits might be less connected to general adiposity or processes in visceral fat depots, respectively.

It could be demonstrated that the DL-based approach [19] is technically robust and efficient. Training the CNN with clinical routine chest CT scans, using both contrast-enhanced and non-contrast-enhanced CT scans from the standardized calcium scan CT protocols, did not diminish its performance. Sørensen Dice Scores did not indicate different performances depending on the use of contrast agent, which was confirmed by manually reviewing the predicted segmentations. While manual chest CT annotation is not feasible in clinical routine, our BCA software predicted full tissue quantification in five to ten seconds, making it more resource-conserving and therefore applicable in clinical practice. The system is able to provide various other data on tissue quantification and their relations to each other (e.g., see Section 2.3), contrary to other DL-based approaches that focus on single biomarkers, e.g., EAT [16,21].

Recent studies have investigated EAT and PAT in a variety of possibly related cardiovascular diseases. Eisenberg et al. and Goeller et al. examined EAT and its relation to MACE, and observed an association between increased EATvol and decreased EATatt and MACE. They described EAT as a cardiovascular risk factor for atherosclerosis and as an independent risk factor for MACE, and found similarly significant differences between EATvol and EATatt when comparing CACS 0 vs. CACS 1–99 category patients [3,16]. Mahabadi et al. support these findings, and indicate different pathways between higher volume of EAT and CAD [27]. As described, we could observe similar differences between these CACS categories with respect to EAT and PAT characteristics in patients. These differences might be important hints for determining which individuals are more likely to develop CAD in future when they currently have no or minor levels of CAC. Judging from our results, neither EAT nor PAT can be used as general reliable surrogate markers for CAC in general since a correlation could not be observed. Still, EAT and PAT assessment might provide potential individual prognostic value when it is examined in early consultation since its accumulation might indicate local pathological processes distinguishable from visceral adiposity, as mentioned above. Also, some sex-based disparities in EAT and PAT characteristics could be observed, which could not be explained in research so far. The role of PAT has to be examined more intensely.

Especially in low- to intermediate-risk patients, automated EAT analysis could provide an additional prognostic marker. In in these patients, the volume of epicardial adipose tissue seems to be associated with an increased risk of cardiovascular events [3,17,33]. Moreover, an EAT analysis can be derived from calcium scoring examinations, which are indicated in this group of patients anyway [23].

As Pickhardt et al. point out, BCA can add great opportunistic value, and even outperform established clinical parameters, for pre-symptomatic risk analysis when deployed to extract individual biometric information from medical images for a variety of clinical indications [20]. The employed DL-based approach provided an efficient biomarker extraction of EAT, PAT and a variety of other tissue biomarkers on a large clinical cohort in a precise manner.

EAT and PAT cannot be used as surrogate markers for CACS, and are not eligible as direct risk markers for CAD. Especially women seem prone to increased coronary calcification related to increased EAT and PAT volume and decreased EAT and PAT radiodensity. Body composition analysis is a potentially practicable method for EAT and PAT biomarker assessment in clinical routine.

We acknowledge some limitations of this study beyond its single-center design. The mean age of menopause [32] is not a strict marker of menopause and should be confirmed by the current hormonal status. Because of the CT protocol’s curtailment of the superior and inferior ends of the heart, PAT was measured in a standardized manner, but could not be measured in total. Because the study design focused on a clinical cohort, precisely characterized patient data on pre-existing diseases, individual risk factors, and outcomes was lacking. However, the conducted study complements other studies performed on study collectives with its findings on imaging data compiled in clinical routine care.

## 5. Conclusions

Our study shows that a fully automated adipose tissue analysis in clinical cardiac CT is feasible and confirms in a large clinical cohort that the volume and attenuation of pericardial adipose tissue is not correlated with coronary calcification score. Against the background of broadly available deep learning-based rapid and reliable tissue quantification and previously described connections between pericardial fatty tissue and cardiovascular events, the routine assessment of this valuable biomarker as a supplementary risk predictor in cardiac CT should be discussed.

## Figures and Tables

**Table 1:** Patient characteristics itemized by age, gender, BMI and Coronary Artery Calcium Score (CACS) categories.

Variable	Numeric Value
No. of examined patients	966 (334 male/632 female)
Age	59.3 ± 9.6 years
BMI (kg/m^2^)	26.9 ± 4.7
CACS 0	526 (134 male/392 female)
CACS 1–99	293 (126 male/167 female)
CACS ≥100	147 (74 male/73 female)

Ethical approval for this study was obtained from the Institutional Review Board (ID: 20-9635-BO).

**Table 2:** Features measured and itemized into gender- and CACS groups (both/male/female) to allow for comparability to other study cohorts.

CACS Category	Mean Value (b/m/f)	Standard Deviation (b/m/f)	95%-CI (b/m/f)
Epicardial Adipose Tissue (EAT)vol (mL)			
0	90.44/108.1/84.44	38.39/46.28/33.29	[87.15, 93.73]/[100.1, 115.91]/[81.13, 87.74]
1–99	108.25/124.35/96.11	44.68/49.71/36.15	[103.12, 113.39]/[115.58, 133.11]/[90.6, 101.63]
≥100	118.25/134.07/102.21	50.61/57.55/36.29	[110.0, 126.5]/[120.74, 147.4]/[93.74, 110.68]
EATatt (HU)			
0	−73.48/−73.25/−73.56	4.72/4.72/4.73	[−73.89, −73.08]/[−74.06, −72.45]/[−74.03, −73.09]
1–99	−74.95/−74.46/−75.32	4.59/4.6/4.57	[−75.48, −74.42]/[−75.28, −73.65]/[−76.02, −74.62]
≥100	−75.08/−75.33/−74.83	5.03/5.41/4.64	[−75.9, −74.26]/[−76.59, −74.08]/[−75.91, −73.742]
Paracardial Adipose Tissue (PAT)vol (mL)			
0	130.77/193.8/109.23	78.67/98.23/56.57	[124.03, 137.51]/[177.02, 210.59]/[103.61, 114.85]
1–99	171.76/223.51/132.72	93.77/99.62/66.55)	[160.99, 182.55]/[205.95, 241.08]/[122.56, 142.89]
≥100	200.46/260.34/139.76	116.6/127.09/61.43	[181.45, 219.47]/[230.89, 289.78]/[125.43, 154.1]
PATatt (HU)			
0	−95.58/−97.78/−94.82	6.02/6.67/5.6	[−96.09, −95.06]/[−98.92, −96.64]/[−95.38, −94.27]
1–99	−98.05/−99.88/−96.66	5.95/5.96/5.57	[−98.73, −97.36]/[−100.93, −98.83]/[−97.51, −95.81]
≥100	−98.44/−100.93/−95.91	6.39/6.62/5.05	[−99.48, −97.4]/[−102.47, −99.4]/[−97.09, −94.73]
BMI (m/kg^2^)			
0	26.49/27.7/26.06	4.8/4.29/4.91	[26.02, 26.96]/[25.49, 26.62]/[26.88, 28.52]
1–99	27.52/28.19/26.93	5.37/4.14/6.22	[26.79, 28.26]/[25.76, 28.16]/[27.36, 29.02]
≥100	26.67/26.44/26.89	4.01/3.28/4.7	[25.9, 27.45]/[25.12, 27.763]/[26.0, 27.78]
Age (years)			
0	53.8/50.13/55.06	9.87/9.92/9.55	[52.96, 54.65]/[48.44, 51.83]/[54.11, 56.01]
1–99	59.89/55.76/63.01	9.42/8.4/8.95	[58.81, 60.97]/[54.27, 57.24]/[61.64, 64.38]
≥100	64.19/61.51/66.91	9.43/9.67/8.4	[62.65, 65.73]/[59.27, 63.75]/[64.95, 68.87]

**Table 3:** Inter-group comparison of CACS groups itemized into gender groups (both/male/female) regarding their significance. The results of Dunn’s Test are stated as not applicable (N/A) when the Kruskal-Wallis test does not reach the significance level.

Feature	Gender (b/m/f)	Kruskal-Wallis Test	Dunn’s Test CACS 0 vs. 1–99	Dunn’s Test CACS 1–99 vs. >100
EATvol (mL)				
	Male	<0.01	<0.01	0.37
	Female	<0.001	<0.001	0.18
	Both	<0.001	<0.001	0.08
EATatt (HU)				
	Male	0.02	0.06	0.35
	Female	<0.001	<0.001	0.40
	Both	<0.001	<0.001	0.98
PATvol (mL)				
	Male	<0.001	<0.01	0.10
	Female	<0.001	<0.001	0.29
	Both	<0.001	<0.001	0.05
PATatt (HU)				
	Male	<0.01	<0.01	0.38
	Female	<0.001	<0.001	0.38
	Both	<0.001	<0.001	0.84
BMI (m/kg^2^)				
	Male	0.18	N/A	N/A
	Female	0.35	N/A	N/A
	Both	0.03	N/A	N/A
Age (years)				
	Male	<0.001	<0.001	<0.001
	Female	<0.001	<0.001	<0.01
	Both	<0.001	<0.001	<0.001

## Data Availability

The data presented in this study are available on request from the corresponding author. The data are not publicly available.

## Abbreviations

BCA	Body Composition Analysis
BMI	Body Mass Index
CAC	Coronary Artery Calcification
CACS	Coronary Artery Calcium Score
CAD	Coronary Artery Disease
CNN	Computed Neural Network
DL	Deep Learning
DT	Dunn’s Post Test
EAT	Epicardial Adipose Tissue
KW-Test	Kruskal-Wallis Test
MACE	Major Adverse Cardiac Event
PAT	Paracardial Adipose Tissue
